# Integrated transcriptomic analysis reveals key regulatory mechanisms of *HIPK2* in osteoarthritis and identifies potential therapeutic target drugs

**DOI:** 10.1097/MD.0000000000049323

**Published:** 2026-06-26

**Authors:** Chunli Bai, Yuxiang Zhang, Bo Gao, Wenli Fan, Gang Ma

**Affiliations:** a Department of General Surgery II, The Second Affiliated Hospital of Baotou Medical College, Inner Mongolia University of Science and Technology, Baotou, Inner Mongolia Autonomous Region, China; b Department of Arthrosis, The Second Affiliated Hospital of Inner Mongolia Medical University, Hohhot, Inner Mongolia Autonomous Region, China.

**Keywords:** extracellular matrix, *HIPK2*, osteoarthritis, single-cell transcriptome sequencing, targeted therapy

## Abstract

Osteoarthritis (OA) is a prevalent chronic joint disorder characterized by cartilage degeneration and extracellular matrix (ECM) remodeling. *HIPK2* is a serine/threonine kinase involved in transcriptional regulation, cell proliferation, and apoptosis. However, its expression pattern and potential relevance in OA remain incompletely understood. We integrated multiple Bulk RNA-seq datasets and single-cell RNA-seq data to characterize *HIPK2* expression in OA and explore its transcriptomic associations. Pathway changes associated with different *HIPK2* expression states were analyzed using AUCell and gene set variation analysis. Weighted gene co-expression network analysis and least absolute shrinkage and selection operator regression were applied to identify candidate genes associated with *HIPK2* and ECM-receptor interaction pathway activity. Molecular docking was further used as an exploratory in silico approach to screen candidate compounds with potential binding affinity to *HIPK2. HIPK2* expression was significantly elevated in OA and was mainly associated with fibrocartilage chondrocytes across both Bulk RNA-seq and single-cell transcriptomic sequencing analyses. Higher *HIPK2* expression was accompanied by lower activity of several ECM-related pathways, especially the ECM-receptor interaction pathway. Weighted gene co-expression network analysis and least absolute shrinkage and selection operator analyses identified *ZNF638*, *DMXL1*, and *VEZT* as candidate genes associated with both *HIPK2* expression and ECM-receptor interaction pathway activity. In addition, molecular docking suggested 6 compounds with potential binding affinity to *HIPK2*, including progesterone, estradiol, ethinyl estradiol, coumestrol, biotin, and phenobarbital. Our integrative transcriptomic analyses suggest that *HIPK2* is associated with OA and may be involved in ECM-related dysregulation in fibrocartilage chondrocytes. *ZNF638*, *DMXL1*, and *VEZT* may represent candidate genes linked to this process. The docking results provide preliminary candidate compounds for future validation. Overall, these findings are hypothesis-generating and require further experimental confirmation in vitro and in vivo.

## 1. Introduction

Osteoarthritis (OA), a globally prevalent chronic disease, is particularly common in the elderly, with prevalence rates ranging from 30% to 40% across different regions and populations. This prevalence increases substantially with age, reaching as high as 85% in individuals over 65 years old.^[1]^ Women, particularly postmenopausal, tend to have a higher incidence of OA than men, especially in the knee and finger joints. Some studies also suggest that OA is more prevalent among urban residents compared to rural populations. The pathogenesis of OA is complex, and research indicates that chondrocytes in OA patients may undergo apoptosis and trigger the degradation of matrix metalloproteinases (MMPs), leading to reduced cartilage thickness and impaired joint function.^[2]^ Additionally, overloading and abnormal biomechanical behavior of the joints accelerate cartilage wear and degradation. Obesity is another critical factor in OA pathogenesis, as it not only increases joint strain but also promotes the release of pro-inflammatory cytokines (e.g., adiponectin, IL-1) from adipose tissue. These cytokines foster inflammation, disrupt cartilage metabolism, and trigger bursal hyperplasia and joint swelling.^[3,4]^ Current treatments for OA primarily focus on pharmacological and surgical approaches. Pharmacological therapies include nonsteroidal anti-inflammatory drugs for pain relief, topical pain creams, and intra-articular hyaluronic acid injections, which enhance joint lubrication and alleviate pain.^[5]^ Surgical interventions range from arthroscopic surgery: used to remove loose bodies and repair cartilage: to total joint replacement for severe cases, particularly in knee and hip OA^[6,7]^ In recent years, emerging therapies such as biologics (e.g., IL-1 antagonists), stem cell therapy, and platelet-rich plasma have also shown potential in clinical practice^[8,9]^ Homeodomain-interacting protein kinase 2 (*HIPK2*) encodes a conserved serine/threonine kinase involved in several cellular processes, including cell proliferation, apoptosis, and transcriptional regulation. *HIPK2* modulates gene expression and TP53 activity, promoting apoptosis through the phosphorylation and indirect acetylation of TP53, thereby inhibiting cell growth.^[10]^ *HIPK2* activation has been strongly associated with apoptosis in colon cancer, making it a potential therapeutic biomarker.^[11–13]^ Dysregulation of *HIPK2* may also contribute to OA pathogenesis, as alterations in its expression could be linked to chondrocyte apoptosis and matrix synthesis, leading to degenerative changes in articular cartilage. Therefore, investigating the role of *HIPK2* in OA and elucidating its underlying molecular mechanisms is of critical importance.

In this study, we integrated Bulk RNA-seq and single-cell transcriptomic sequencing (scRNA-seq) data to characterize the expression pattern of *HIPK2* in OA and to explore its potential transcriptomic associations with chondrocyte subpopulations and extracellular matrix (ECM)-related signaling pathways. In addition, we used WGCNA and least absolute shrinkage and selection operator (LASSO) regression to identify candidate genes associated with *HIPK2*-related pathway changes and performed molecular docking as an exploratory strategy to screen compounds with potential binding affinity to *HIPK2*. We emphasize that this study is based on computational and transcriptomic analyses; therefore, the findings should be regarded as hypothesis-generating rather than evidence of direct causality.

## 2. Methods

### 2.1. Data sources used for analysis

This study was based exclusively on publicly available, de-identified datasets retrieved from the GEO database. No new human participants were recruited, no new human samples were collected, and no identifiable personal information was involved. Therefore, ethical approval and informed consent were not required for this secondary analysis of public data. The inclusion criteria encompassed mRNA expression profiles generated through array-based or high-throughput sequencing platforms, ensuring the inclusion of *HIPK2* expression, as well as mRNA expression profiles obtained using 10x Genomics sequencing. All tissue samples were derived from human subjects and were associated with OA pathology. In total, 7 Bulk RNA-seq datasets were included: GSE162691, GSE192982, GSE176223, GSE75432, GSE99662, GSE188374, GSE135854, and GSE255460, along with one scRNA-seq dataset.

### 2.2. Data preprocessing

The 7 collected Bulk RNA-seq datasets were merged into an integrated expression matrix using the merge function in R. Batch effects were addressed using the R package “limma,”^[14]^ which was employed for batch effect correction. The effectiveness of batch removal was assessed by visualizing the integrated Bulk RNA-seq results through principal component analysis (PCA) and t-distributed stochastic neighbor embedding analyses. For the scRNA-seq data, the R packages “Seurat”^[15]^ and “harmony”^[16]^ were utilized for standard single-cell processing and batch correction. Quality control criteria included a minimum cell count of 5, a minimum gene count of 500, a mitochondrial gene proportion of <10%, and a red blood cell proportion of less than 1%. The first 30 dimensions from PCA downscaling were used for batch correction via harmony, with cell clustering at a resolution of 0.6. Cell annotation was based on well-defined cell types from the original data, and the final single-cell information was visualized using UMAP plots.

### 2.3. Exploring the relationship between HIPK2 and immune cell infiltration

The relative enrichment of immune-related signatures was estimated from the integrated Bulk RNA-seq data using the R package “gene set variation analysis (GSVA).” A total of 28 immune cell-related gene sets were selected based on prior studies.^[17]^ Samples were stratified into high- and low-*HIPK2* expression groups according to the median *HIPK2* level. Differences in estimated immune-related signature scores between the 2 groups were then compared. These analyses were intended to provide a transcriptome-based estimation of immune-associated patterns rather than direct quantification of immune cell abundance or activity.

### 2.4. Detection of HIPK2 specific expression in scRNA-seq

The differential expression of *HIPK2* between the OA and control groups was initially compared based on the cell fractionation data provided in the raw dataset. Next, *HIPK2* expression was analyzed across different cell subtypes within the dataset. Finally, single-cell data from the OA group were extracted and analyzed to compare *HIPK2* expression in specific cell subpopulations.

### 2.5. Cibersort inverse convolution calculates cell abundance in bulk RNA-seq

For each cell type, the top 10 differentially expressed marker genes were identified using filtering criteria of minimum percentage = 0.5 and logFC = 0.5, retaining markers upregulated in the corresponding cell type. The average expression profiles of these marker genes from OA scRNA-seq cell subpopulations were used as a reference input for CIBERSORT analysis^[18]^ to estimate the relative abundance of cell subpopulations in each Bulk RNA-seq sample. Because this deconvolution procedure depends on the selected marker genes and reference profiles, the results should be interpreted as computational estimates rather than direct measurements of cell composition.

### 2.6. Pseudotime analysis of the regulatory role of HIPK2 in differentiation

The preFC and FC cell information from OA samples was separately extracted, and pseudotime trajectories for both cell types were analyzed using the R package “monocle.”^[19]^ This analysis was conducted to construct the differentiation trajectories of preFC and FC cells. A heatmap was generated to visualize *HIPK2* expression along the differentiation pathways of both cell types, allowing us to assess whether *HIPK2* plays a regulatory role in their development and differentiation.

### 2.7. Investigating alterations in pathway activity triggered by HIPK2 dysregulation

FC cells from OA samples in the scRNA-seq data were first individually extracted to identify those expressing *HIPK2*. These cells were then categorized into high and low expression groups based on the median *HIPK2* expression level. The FindAllMarkers function in the Seurat package was employed to calculate differentially expressed genes between the *HIPK2* high expression and low expression groups. The kyoto encyclopedia of genes and genomes (KEGG) and gene ontology (GO) pathways for these differential genes were enriched using the R package “clusterProfiler.” The gene sets corresponding to the enriched pathways were extracted, and AUCell scores for these pathways were calculated for each cell using the R package “AUCell.” The R package “limma” was then utilized to determine the fold difference in AUCell scores between the *HIPK2* high and low expression groups, screening for pathways with a *P*-value of <0.05. Additionally, GSVA scores for the differentially gene-enriched pathways were calculated in the Bulk RNA-seq cohort. The GSVA scores were also analyzed using the ‘limma’ package to assess the multiplicity of differences between the *HIPK2* high and low expression groups, with pathways having a *P*-value < 0.05 being screened.

### 2.8. Screening of scRNA-seq and Bulk RNA-seq co-ascending and descending pathways

KEGG and GO pathways with significant differences in AUCell and GSVA scores from the scRNA-seq and Bulk RNA-seq analyses were extracted separately, and their intersections were evaluated using the R package “VennDiagram.” Pathways with a logFC <0 and <0 in both datasets were further screened to identify consistently upregulated and downregulated signaling pathways. Venn diagrams were employed to visualize the results of this intersection analysis, while box-and-line plots were used to illustrate the differences in signaling pathway activity between the 2 groups.

### 2.9. Analysis of HIPK2 high relevance signaling pathway and Hub genes

Bulk RNA-seq data were analyzed using weighted gene co-expression network analysis (WGCNA)^[20]^ through the R package “WGCNA,” which clusters genes into color-coded modules based on their co-expression patterns. The GSVA scores of the synapomorphy signaling pathways identified in the previous step were then correlated with these gene modules. After determining the gene modules that exhibited the highest correlation with the phenotype, Hub genes that were strongly correlated with both *HIPK2* and pathway activity (GS > 0.2, MM > 0.8) were further screened. Differences in the expression of Hub genes between high and low *HIPK2* expression subgroups were visualized using box-and-line plots. Pearson correlations were computed for Hub genes with *HIPK2* and pathway activity scores to elucidate their relationships. Additionally, LASSO regression was employed to identify Hub genes with the strongest association to the signaling pathways, providing new insights into the specific regulatory molecules of *HIPK2*.

### 2.10. Screening for potential target drugs for HIPK2

The Comparative Toxicogenomics Database (CTD)^[21]^ was used to identify compounds reported to be associated with *HIPK2*. Structural information for these compounds was obtained from the PubChem database, while the protein structure used for docking analysis was retrieved from public structural resources. Molecular docking was performed using AutoDock as an exploratory in silico screening method to estimate potential binding affinity between candidate compounds and *HIPK2*. PyMOL was used for visualization. These docking results were used only for preliminary candidate prioritization and do not constitute evidence of biological efficacy, target specificity, or therapeutic benefit in OA.

### 2.11. Statistical analysis

R version 4.4.1 was used for statistical analysis. *T*-tests were used for statistical tests between different groups, with *P*-value <.05 indicated statistical significance (*P < .05; **P < .01; ***P < .001; ****P < .0001). Pearson Correlation Analysis was used to calculate correlations.

## 3. Results

### 3.1. Immune cell specificity of HIPK2

**Figure 1.**
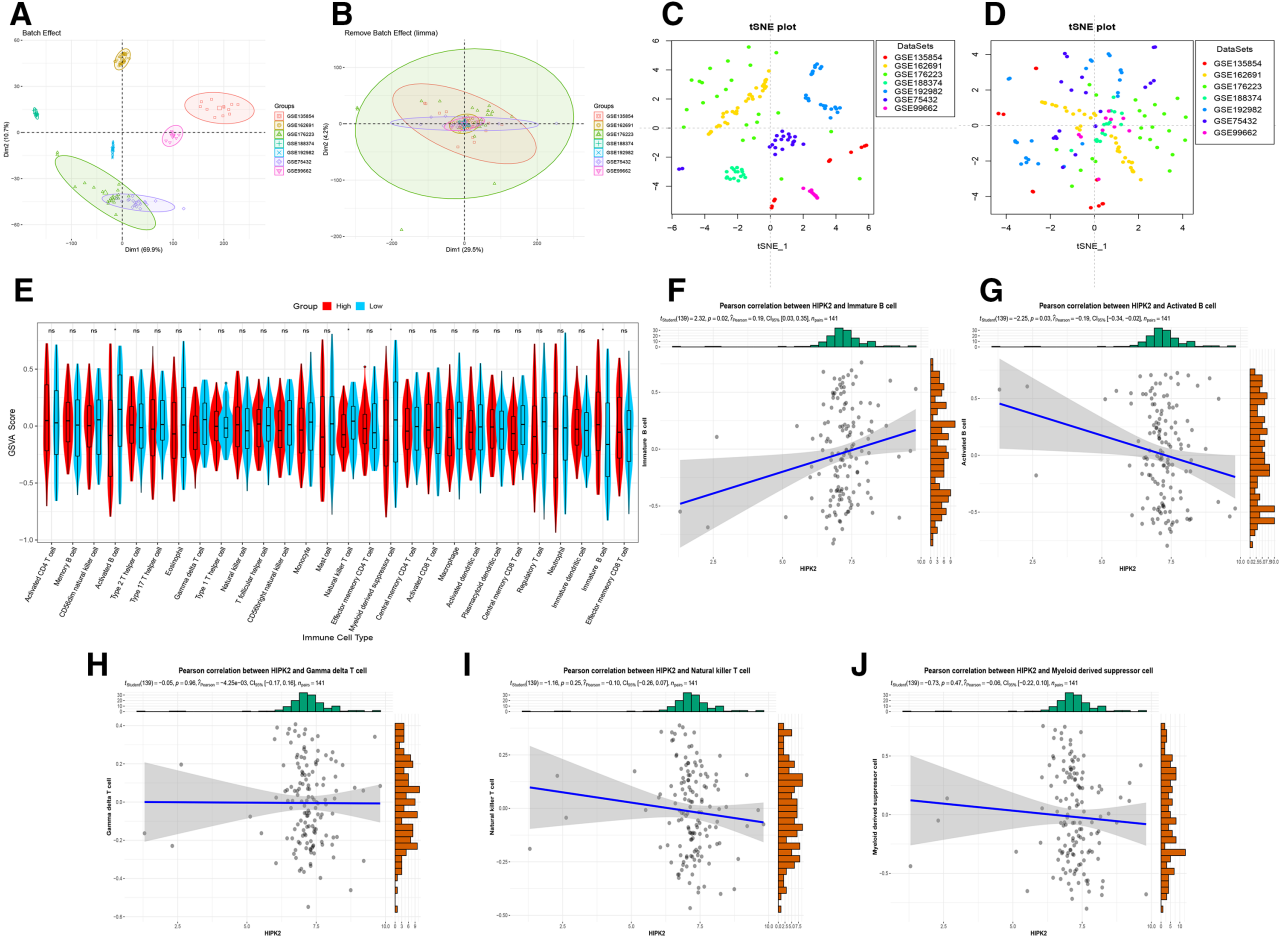
Integrated analysis of bulk RNA-seq data. **(A**) Principal component analysis (PCA) of the integrated bulk RNA-seq data, illustrating the differences before de-batching treatment. **(B**) PCA analysis after de-batching treatment, showing improved clustering. **(C**) t-SNE downscaling is used to compare differences before de-batching. **(D**) t-SNE downscaling post de-batching treatment, indicating enhanced separation. **(E**) Immune infiltration analysis of the Bulk RNA-seq cohort, highlighting differences in immune cell infiltration between high and low *HIPK2* expression subgroups. **(F**) Pearson correlation analysis of *HIPK2* expression levels with Immature B cells. **(G**) Pearson correlation analysis of *HIPK2* expression levels with Activated B cells. **(H**) Pearson correlation analysis of *HIPK2* expression levels with Gamma delta T cells. **(I**) Pearson correlation analysis of *HIPK2* expression levels with Natural Killer T cells. **(J**) Pearson correlation analysis of *HIPK2* expression levels with myeloid derived suppressor cells. Statistical significance is denoted as follows: *P < .05; **P < .01; ***P < .001; ****P < .0001. t-SNE = t-distributed stochastic neighbor embedding.

Although the etiology of OA is primarily attributed to mechanical injury and degenerative changes, recent studies have highlighted the role of the immune system: particularly innate immune cells and low-grade inflammation: in the pathogenesis and progression of OA. The inflammatory response significantly contributes to joint damage, cartilage degeneration, and pain. To investigate whether *HIPK2* is involved in regulating the inflammatory response of immune cells, we conducted an immune infiltration analysis using integrated Bulk RNA-seq data. Before this analysis, we integrated 7 Bulk RNA-seq datasets and removed batch effects. We compared the data distribution before and after batch effect removal using PCA and t-distributed stochastic neighbor embedding analyses (Figs. 1A-D), which demonstrated that the integrated data, after batch effect removal, could be regarded as a dataset for further analysis. Subsequently, GSVA was employed to calculate the infiltration abundance of 28 immune cell types. The samples were then grouped based on *HIPK2* expression levels to observe differences in immune cell infiltration between the *HIPK2* high-expression and low-expression groups. As illustrated in Figure 1E, the *HIPK2* high-expression group exhibited a higher abundance of immature B cells, indicating an upregulation of *HIPK2* expression in these cells. Conversely, activated B cells, gamma delta T cells, natural killer T cells, and myeloid-derived suppressor cells showed greater abundance in the *HIPK2* low-expression group, suggesting downregulation of *HIPK2* in these immune cell types. Further Pearson correlation analyses between *HIPK2* and these 5 immune cell types (Figs. 1F-J) indicated that *HIPK2* is more abundant in immature immune cells, while its expression is downregulated in mature immune cells performing their immune functions. These findings suggest that *HIPK2* expression is associated with differences in estimated immune-related signatures; however, the results do not establish a direct role for *HIPK2* in immune-cell regulation or inflammatory responses. Therefore, we next focused on its expression pattern in chondrocyte populations.

### 3.2. Chondrocyte specificity of HIPK2

**Figure 2.**
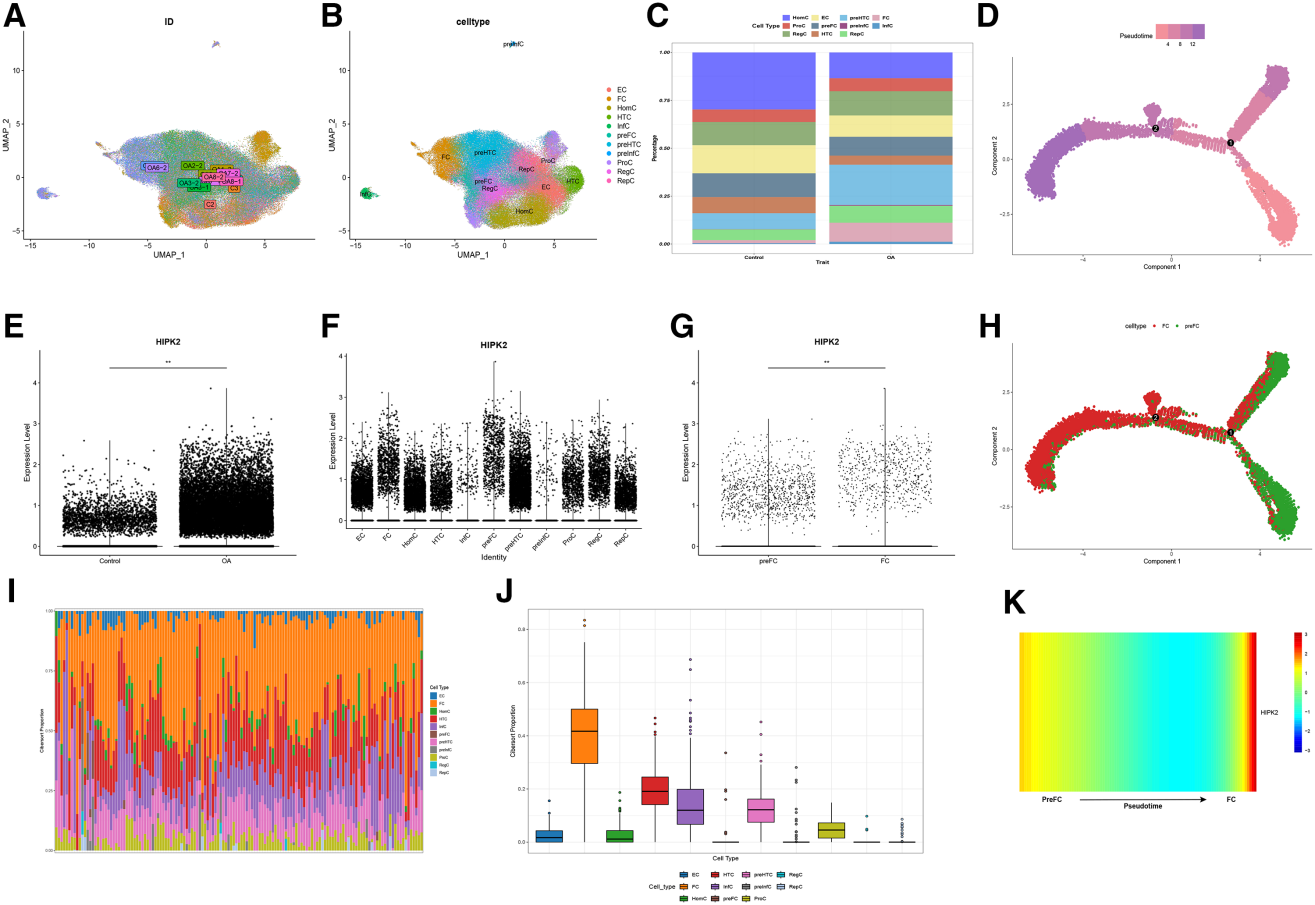
scRNA-seq data for multidimensional analysis of *HIPK2* expression differences. **(A**) UMAP plot visualizing the distribution of sample cells after cleaning the scRNA-seq data. **(B**) UMAP plots annotated with preexisting cell type information. **(C**) Comparison of cell type differences between OA and normal samples, highlighting distinct distributions. **(D**) Pseudotime differentiation axis constructed from preFC and FC cells, illustrating their developmental trajectory. **(E**) Comparison of *HIPK2* expression levels between OA and normal samples. **(F**) Differences in *HIPK2* expression across various cell types. **(G**) Comparison of *HIPK2* expression levels between OA samples seeded with FC cells and preFC cells. **(H**) Distribution differences of preFC and FC cells on the pseudotime differentiation axis. **(I**) CIBERSORT analysis calculated the abundance of different chondrocyte types in Bulk RNA-seq, visualized as histograms. **(J**) Box plots representing the abundance of different chondrocytes calculated by CIBERSORT. **(K**) Heatmaps displaying the expression levels of *HIPK2* mapped to the pseudotime axis of preFC and FC cells. OA = Osteoarthritis.

To investigate the expression differences of *HIPK2* in chondrocytes between OA and normal samples, we performed secondary analyses based on previous single-cell studies of OA. Our analysis involved reprocessing single-cell expression profiles to remove batch effects, resulting in 128,703 high-quality cells (Fig. 2A). To accurately restore the original cell types, we utilized the cell type annotation information provided by the original authors. In total, we identified 11 distinct types of chondrocytes in the reclustered cell taxa (Fig. 2B): effector chondrocytes, fibrocartilage chondrocytes (FC), homeostatic chondrocytes, hypertrophic chondrocytes, inflammatory chondrocytes, prefibrocartilage chondrocytes (preFC), prehypertrophic chondrocytes, preinflammatory chondrocytes, proliferative chondrocytes (proC), regulatory chondrocytes, and reparative chondrocytes. Notably, OA samples exhibited a higher abundance of preHTC and FC cells, alongside a reduction in HomC cells (Fig. 2C). Next, we compared the expression of *HIPK2* across different chondrocyte subgroups. The analysis revealed significantly higher levels of *HIPK2* expression in OA samples compared to normal samples (Fig. 2E). When examining specific chondrocyte types, we found that *HIPK2* expression was particularly elevated in preFC and FC cells (Fig. 2F). Further comparison of *HIPK2* expression in preFC and FC within OA samples demonstrated that *HIPK2* was expressed at higher levels in FC cells (Fig. 2G). We then extracted preFC and FC cells to conduct pseudotime analysis (Fig. 2D), which indicated that preFC cells were closer to the early stages of differentiation, while FC cells were positioned nearer to the late stages of differentiation (Fig. 2H), consistent with expected differentiation patterns. Based on the constructed pseudotime axes, we assessed the differences in *HIPK2* expression during the differentiation of preFC and FC cells. The pseudotime heatmap (Fig. 2K) illustrated a marked increase in *HIPK2* expression at the end of differentiation, particularly in mature FC cells. Moreover, the specificity of *HIPK2* expression in FC cells was corroborated by our integrated Bulk RNA-seq data. We calculated the cellular abundance of integrated data using the CIBERSORT algorithm, and visualization results indicated varying abundances of different chondrocyte types in OA, with FC showing notably high abundance (Figs. 2I-J). In summary, FC cells were significantly enriched in OA samples, and *HIPK2* was abnormally highly expressed in FC cells within OA, suggesting potential roles of *HIPK2* in regulating chondrocyte function and differentiation. Further analyses focusing on FC in OA will be conducted to elucidate these regulatory functions.

### 3.3. Pathway activity changes associated with different HIPK2 expression states

**Figure 3.**
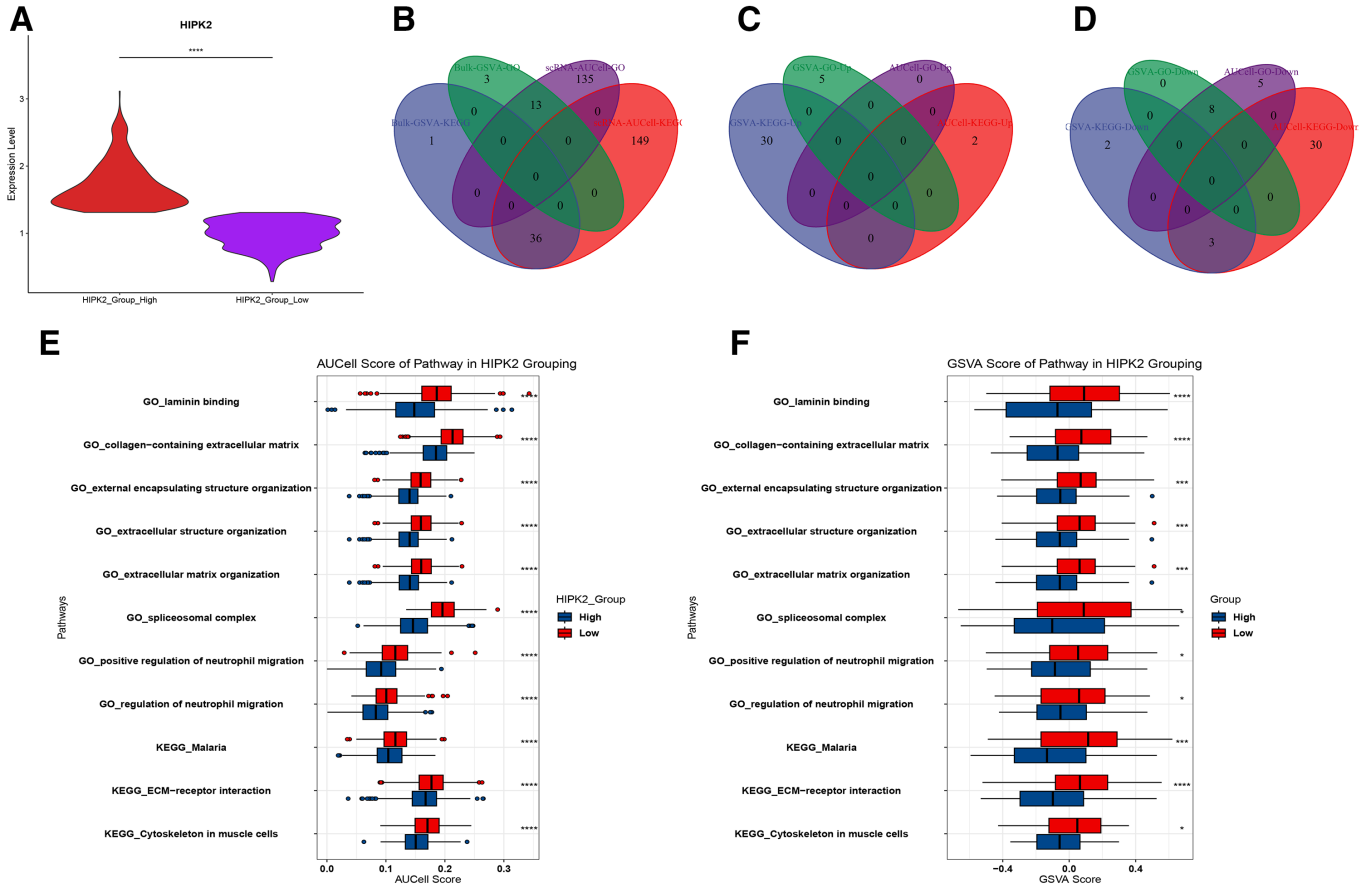
Simulated alterations in FC cell pathway activity triggered by *HIPK2* dysregulation. **(A**) FC cells were categorized into high and low *HIPK2* expression groups based on the median *HIPK2* level. **(B**) Differences in AUCell scores and GSVA scores between high and low *HIPK2* groups were analyzed, with pathways identified based on a *P*-value threshold of <.05. **(C**) Pathways where both AUCell scores and GSVA scores were significantly up-regulated. **(D**) Pathways where both AUCell scores and GSVA scores were significantly down-regulated. **(E**) Visualization of AUCell scores for the screened KEGG and GO signaling pathways, highlighting differences in pathway activity between the high and low *HIPK2* groups. **(F**) Visualization of GSVA scores for the same screened KEGG and GO signaling pathways, comparing activity differences between the high and low *HIPK2* groups. (*P < .05; **P < .01; ***P < .001; ****P < .0001). GO = gene ontology, GSVA = gene set variation analysis, KEGG = kyoto encyclopedia of genes and genomes.

To further investigate the signaling pathways mediating the regulatory role of *HIPK2* in fibrocartilage (FC) cells, we categorized FC cells from single-cell data into *HIPK2* high-expression and low-expression groups based on their expression levels (Fig. 3A). We then identified the KEGG and GO pathways associated with these 2 groups. Next, we calculated AUCell scores for the single-cell data and GSVA scores for the Bulk RNA-seq data, assessing the multiplicity of difference based on the pathway scores to screen for significantly different pathways. We selected signaling pathways with a *P*-value <.05 for both expression levels for further intersection analysis (Fig. 3B), yielding 13 intersecting pathways for GO and 36 for KEGG. Considering the technical differences between scRNA-seq and Bulk RNA-seq, we further refined our analysis to identify co-ascending and co-degrading signaling pathways. This involved examining pathways where the multiplicity of difference was either consistently positive or negative. The results, depicted in Figure 3C-D, revealed no co-ascending pathways; however, we identified a total of 11 co-degrading pathways. These included KEGG pathways such as Malaria, ECM-receptor interaction, and Cytoskeleton in muscle cells, along with GO pathways related to laminin binding, collagen-containing ECM organization, extracellular structure organization, spliceosomal complex, positive regulation of neutrophil migration, and regulation of neutrophil migration (Figs. 3E-F). In summary, when *HIPK2* expression was upregulated, the activity of signaling pathways related to ECM regulation and neutrophil migration was downregulated. This suggests that *HIPK2* plays a role in mediating ECM regulation in FC cells, subsequently influencing microenvironmental changes.

### 3.4. Signalling pathways and regulatory genes highly associated with HIPK2 in OA

**Figure 4.**
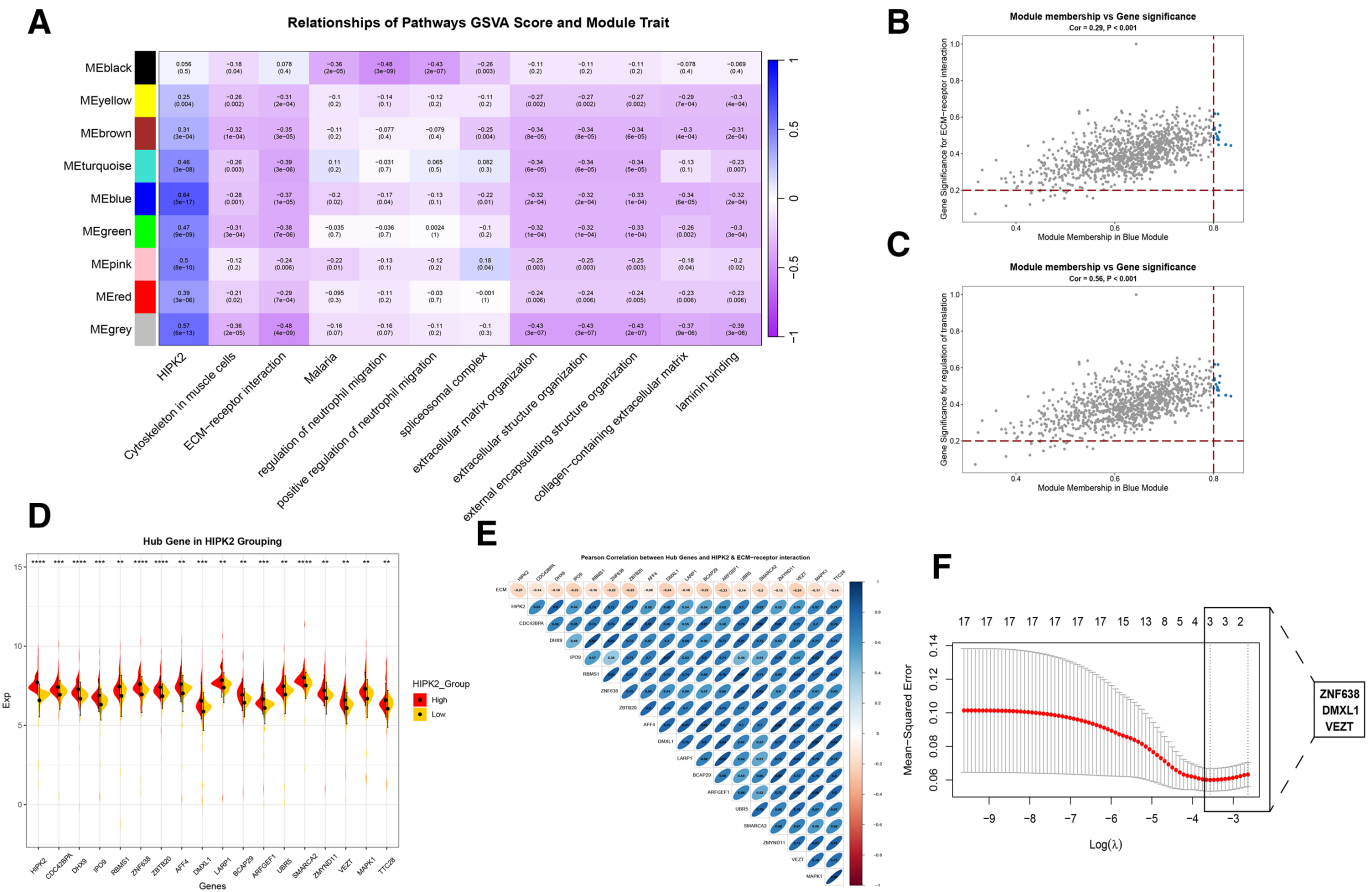
WGCNA analysis based on bulk RNA-seq data. **(A**) Pearson correlation analysis between different colored gene modules identified through WGCNA and *HIPK2* expression, as well as signaling pathway GSVA scores. **(B**) Identification of hub genes within the blue gene module associated with ECM-receptor interaction. **(C**) Identification of hub genes within the blue gene module associated with *HIPK2*. **(D**) Comparison of expression levels of merged hub genes between high and low *HIPK2* subgroups. **(E**) Pearson correlation analysis of merged hub genes with *HIPK2* expression and ECM-receptor interaction activities, respectively. **(F**) LASSO regression analysis of hub genes significantly associated with ECM-receptor interaction activity. (*P < .05; **P < .01; ***P < .001; ****P < .0001). ECM = Extracellular matrix, GSVA = gene set variation analysis, LASSO = least absolute shrinkage and selection operator, WGCNA = Weighted gene co-expression network analysis.

To further elucidate the signaling pathways that are highly correlated with *HIPK2* and the genes it regulates, we performed WGCNA analysis on the integrated Bulk RNA-seq cohort. Following the identification of different colored gene modules, we utilized the GSVA score matrices of the aforementioned 11 pathways as phenotypic information. We then conducted correlation analysis between these phenotypes and the gene modules. As illustrated in Figure 4A, the blue gene module exhibited a strong positive correlation with *HIPK2*, with a correlation coefficient of 0.64. In contrast, the ECM-receptor interaction pathway showed a significant negative correlation with the blue module, with a coefficient of −0.37. Additionally, the gray gene module also demonstrated a negative correlation with ECM-receptor interaction (correlation coefficient of −0.48) and a positive correlation with *HIPK2* (correlation coefficient of 0.57). However, since the gray gene module did not yield any Hub genes that met the screening criteria during subsequent analyses, we focused solely on the blue gene module for Hub gene screening. We calculated the correlation of gene co-expression within the blue gene module with the *HIPK2* and ECM-receptor interaction phenotypes, ultimately identifying 17 Hub genes (Figs. 4B-C). When comparing the differential expression of these Hub genes in the high and low *HIPK2* subgroups (Fig. 4D), we observed that all Hub genes were upregulated in the *HIPK2* high expression group. This pattern suggests that high *HIPK2* expression is accompanied by increased expression of these hub genes and reduced ECM-receptor interaction pathway activity. However, the present analyses do not establish that *HIPK2* directly regulates these genes or pathway changes. Further Pearson correlation analysis revealed that *DHX9* exhibited the highest positive correlation with *HIPK2* (correlation coefficient of 0.8), while *DMXL1* and *VEZT* showed the highest negative correlation with ECM-receptor interaction (both with a coefficient of −0.24) (Fig. 4E). To investigate the relationship between Hub genes and ECM-receptor interaction further, we employed LASSO regression. The results indicated that *ZNF638*, *DMXL1*, and *VEZT* were most strongly associated with ECM-receptor interaction (Fig. 4F), suggesting that *HIPK2* may downregulate ECM-receptor interaction by modulating the expression levels of these 3 genes.

### 3.5. Potential targeted drugs for down-regulation of HIPK2

**Figure 5.**
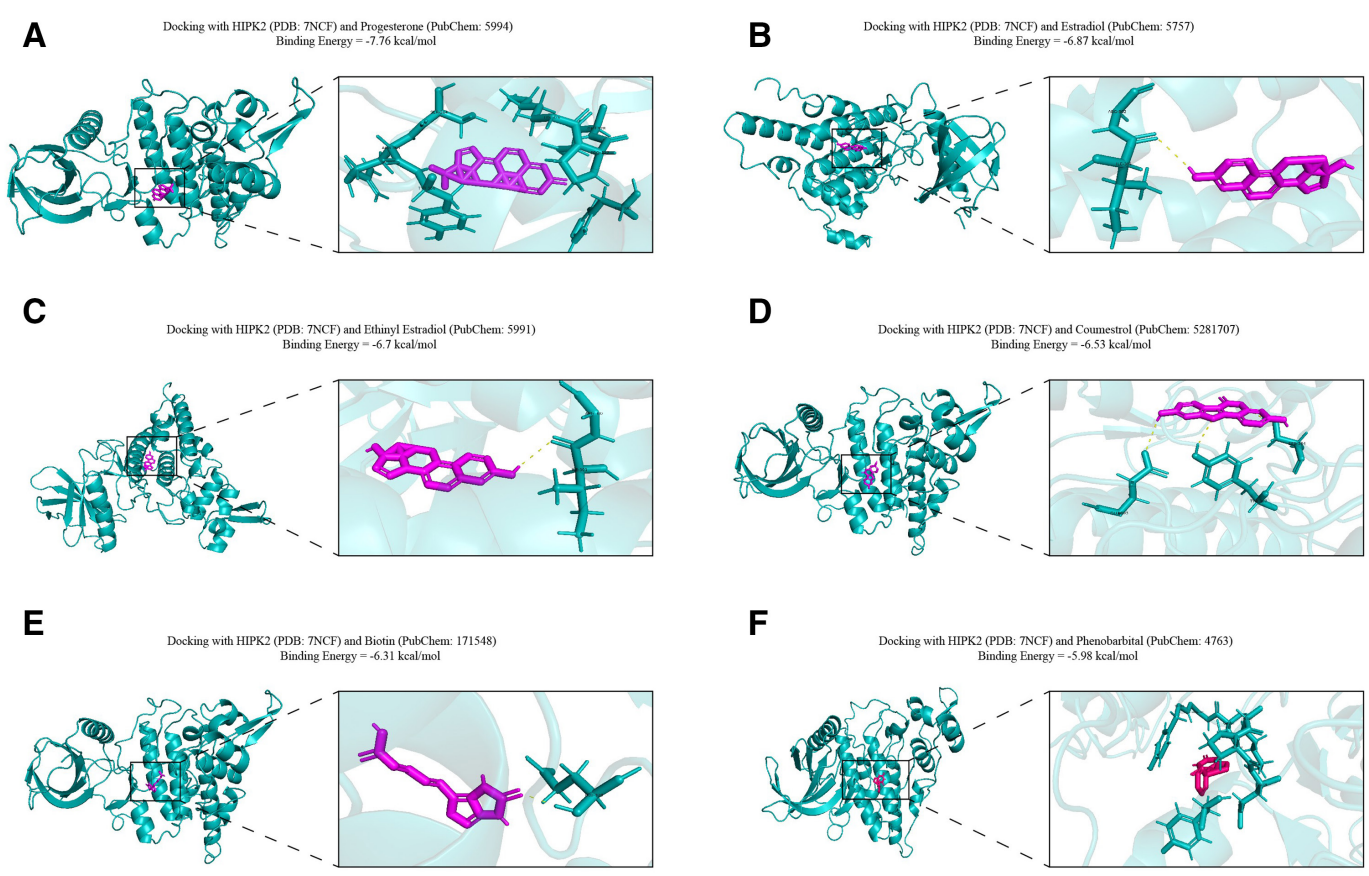
Molecular docking diagrams of *HIPK2* with potential target drugs. **(A**) Docking effect plot of *HIPK2* with Progesterone. **(B**) Docking effect diagram of *HIPK2* with Estradiol. **(C**) Docking effect diagram of *HIPK2* with Ethinyl Estradiol. **(D**) Docking effect diagram of *HIPK2* with Coumestrol. **(E**) Docking effect diagram of *HIPK2* with Biotin. **(F**) Docking effect diagram of *HIPK2* with Phenobarbital.

Based on the findings, further screening of *HIPK2*-targeted drugs could facilitate the proposal of targeted therapies for OA. We retrieved a set of compounds capable of targeting and reducing *HIPK2* expression from the CTD Database as ligands, and we downloaded the protein structure of *HIPK2* as the receptor. Molecular docking was then conducted using AutoDock, followed by the screening of docking binding energies and compound properties, which led to the identification of 6 potential target drugs. Sorting the docking results in descending order of binding energy revealed that the top 4 drugs with the highest binding affinities are all hormone-related medications. Notably, the compound with the highest docking binding energy to *HIPK2* was progesterone, exhibiting a binding energy of −7.76 kcal/mol (Fig. 5A). Interestingly, no hydrogen-bonding binding sites were observed for progesterone, suggesting that it may interact with *HIPK2* through alternative mechanisms. Following progesterone, estradiol displayed a docking binding energy of −6.87 kcal/mol (Fig. 5B), with binding sites identified at isoleucine 301 and arginine 302. The third compound, ethinyl estradiol, had a docking binding energy of −6.7 kcal/mol (Fig. 5C), with binding sites mirroring those of estradiol at isoleucine 301 and arginine 302. The fourth compound, coumestrol, yielded a docking binding energy of −6.53 kcal/mol (Fig. 5D), with binding sites at serine 364, tyrosine 367, and glutamic acid 393. Additionally, vitamin B7 (biotin) exhibited a docking binding energy of −6.31 kcal/mol (Fig. 5E), with a binding site located at glutamate 328. Lastly, phenobarbital demonstrated a docking binding energy of −5.98 kcal/mol (Fig. 5F), and similarly to progesterone, no hydrogen-bonding binding sites were identified, indicating the possibility of alternative binding modes. These findings provide a valuable foundation for exploring targeted therapies aimed at modulating *HIPK2* activity in OA.

## 4. Discussion

OA is a prevalent chronic disease affecting the middle-aged and elderly population, primarily caused by mechanical injury, specifically damage to chondrocytes due to excessive joint tissue loading.^[22]^ Recent studies have indicated that the activation of the Wnt signaling pathway in OA is associated with chondrocyte degeneration and the formation of bone spurs.^[23]^ The *TGF-β* signaling pathway has a dual role in chondrocytes, providing protective effects that maintain chondrocyte activity in healthy joints. However, in the context of OA, the *TGF-β* pathway exacerbates the disease by promoting abnormal remodeling of subchondral bone.^[24]^ Additionally, the Notch signaling pathway is implicated in cartilage differentiation and regeneration, with its dysregulation contributing to the pathological processes of OA. The pathogenicity of OA is closely linked to the degradation of the ECM, where MMPs, such as MMP-13 and ADAMTS-5, play critical roles in catabolizing collagen and proteoglycans in the cartilage matrix. These enzymes often exhibit aberrant methylation patterns in OA, leading to increased ECM degradation.^[25]^ Moreover, ECM degradation is associated with various pro-inflammatory cytokines. Cytokines like IL-1β, TNF-α, and IL-6 significantly contribute to OA pathogenesis by promoting cartilage ECM degradation and inducing chondrocyte apoptosis through the activation of relevant signaling pathways. Notably, the NF-κB pathway is central to the activity of these cytokines, with its upregulation closely linked to inflammatory responses and tissue damage.^[26]^ Noncoding RNAs, particularly microRNAs (miRNAs), also play crucial roles in OA pathogenesis. miRNAs can regulate ECM metabolism, apoptosis, and inflammation by modulating the expression of specific target genes. For instance, miR-140 is highly expressed in normal chondrocytes and protects cartilage by inhibiting the expression of MMP-13. Its downregulation in OA may contribute to cartilage degeneration.^[27]^ The pathogenesis of OA is evidently complex, necessitating future studies that integrate genomic, epigenetic, transcriptomic, proteomic, and metabolomic data to elucidate the intricate mechanisms underlying the disease. Furthermore, research into molecular and epigenetic markers may provide new avenues for early diagnosis and personalized treatment strategies for OA. The development of targeted drugs addressing epigenetic alterations in OA may emerge as a critical direction for future therapies. *HIPK2* has an extensive research background at both molecular and epigenetic levels, particularly concerning cancer, apoptosis, gene regulation, and its potential roles in OA.^[28]^ *HIPK2* is crucial in the DNA damage response and tumor suppression, primarily by phosphorylating specific transcription factors that regulate gene transcription, notably phosphorylating p53, which shifts the signaling from cell cycle arrest to apoptosis.^[11]^ Evidence suggests that *HIPK2* is linked to epigenetic regulation associated with DNA damage, regulating chromatin conformation and promoting the transcription of specific genes like p53 through interactions with histone acetylases.^[29]^ Several studies have indicated that *HIPK2* interacts with specific miRNAs, influencing cell proliferation and apoptosis by modulating *HIPK2* expression and affecting the cell cycle, apoptosis, and development.^[30]^ Unfortunately, our literature search did not yield any studies directly addressing *HIPK2* in OA. Given the multifaceted regulatory mechanisms of *HIPK2* and the complex pathogenesis of OA, we hypothesize that there may be significant associations between *HIPK2* and OA pathogenesis. To investigate this, we performed a joint analysis of multiple integrated Bulk RNA-seq datasets alongside single-cell RNA-seq data to deeply explore the regulatory mechanisms of *HIPK2* in OA, aiming to elucidate the role of *HIPK2* in OA pathogenesis and propose new therapeutic options targeting this pathway.

Initially, we retrieved 7 Bulk RNA-seq datasets of OA samples with *HIPK2* expression from the GEO database, along with one scRNA-seq dataset that included both normal and OA samples. Through de-batching methods and integration effect assessments, we successfully integrated the anti Bulk RNA-seq datasets for joint analysis. In the immune infiltration analysis, we observed that *HIPK2* was highly positively correlated with immature B cells, while showing no significant negative correlation with mature immune cells. This suggests that *HIPK2* is less associated with inflammatory responses induced by immune cells in promoting OA pathogenesis, indicating a need to focus on its relationship with chondrocytes. To this end, we analyzed the scRNA-seq data regarding *HIPK2* expression in normal and OA samples. The results revealed that *HIPK2* expression levels were upregulated in OA and were highly correlated with FC cells. FC cells, as a major cell type in cartilage, are specialized chondrocytes enriched in collagen fibers. They are highly resilient, resistant to compression, and primarily produce type II collagen and significant amounts of type I collagen in the ECM, which grants chondrocytes strong compressive and tensile properties.^[31,32]^ Next, we grouped FC cells into high and low *HIPK2* expression groups and analyzed the signaling pathways triggered by *HIPK2* dysregulation. We further assessed the altered activity of these signaling pathways through a combination of Bulk RNA-seq and scRNA-seq data. An in-depth analysis using WGCNA revealed that *HIPK2* was highly correlated with ECM-receptor interaction in FC cells. Notably, *ZNF638*, *DMXL1*, and *VEZT* exhibited strong correlations with both *HIPK2* and the ECM-receptor interaction pathway. *ZNF638*, a member of the zinc finger protein family, is known for its DNA-binding capacity and role in transcriptional regulation. It participates in various biological processes, including cell differentiation, development, and lipid metabolism.^[33,34]^ *DMXL1*, part of the DMX-like family, is involved in synaptic plasticity and nervous system regulation.^[35]^ Additionally, *DMXL1* plays a critical role during embryonic development and is associated with endosomal transport-related pathways.^[36]^ *VEZT* encodes a transmembrane protein essential for cell adhesion and motility, contributing to intercellular junctions and the formation of adhesion complexes, particularly in epithelial cells.^[37]^ Notably, *VEZT* has been shown to mediate significant activity in regulating seizures.^[38]^. In summary, these analyses suggest that *HIPK2* is specifically upregulated in OA, leading to the concurrent upregulation of *ZNF638*, *DMXL1*, and *VEZT* activities. This, in turn, promotes a decrease in the activity of the ECM-receptor interaction signaling pathway, facilitating ECM degradation and contributing to the pathogenesis of OA.

In contrast, screening for targeted drugs that down-regulate *HIPK2* could help alleviate ECM degradation and reduce the risk of OA. To this end, we combined the CTD Database with molecular docking technology to identify 6 potential target drugs with high affinity for *HIPK2*: Progesterone, Estradiol, Ethinyl Estradiol, Coumestrol, Biotin, and Phenobarbital. Notably, the top 4 drugs with the strongest interactions with *HIPK2* were all estrogenic drugs, which are commonly used in clinical practice for OA treatment.^[39]^ This strengthens our belief that *HIPK2* is a key molecule in the pathogenesis of OA and that its potential targeted therapy is of great value. Progesterone is a naturally occurring steroid hormone predominantly secreted by the corpus luteum of the ovary, with smaller amounts produced by the adrenal glands and placenta.^[40]^ It regulates gene expression by binding to progesterone receptors in the nucleus, affecting the menstrual cycle, pregnancy maintenance, and breast development^[41]^。Clinically, progesterone is utilized to treat menstrual irregularities, abnormal uterine bleeding, and to provide luteal support in in vitro fertilization. It is often combined with other estrogens for hormone replacement therapy to prevent endometrial hyperplasia in menopausal women.^[42]^ Estradiol, the predominant and most active estrogen, is synthesized in tissues such as ovarian granulosa cells and the adrenal cortex. Estradiol, the predominant and most active estrogen, is synthesized in tissues such as ovarian granulosa cells and the adrenal cortex.^[43]^ It regulates gene transcription by binding to estrogen receptors, promoting reproductive and bone development, and regulating metabolism. Estradiol is commonly prescribed in hormone replacement therapy to treat menopausal symptoms, prevent osteoporosis, and manage certain types of breast and prostate cancers.^[44]^ However, long-term use may increase the risk of endometrial cancer, breast cancer, and blood clots. Ethinyl Estradiol, a synthetic estrogen, differs from the aforementioned drugs in that it has greater oral bioavailability and is frequently used in oral contraceptives. It functions by inhibiting gonadotropin secretion, preventing ovulation, and altering the nature of cervical mucus.^[45]^ Ethinyl Estradiol is also used to treat female reproductive system dysfunction and is often combined with progestins in hormone replacement therapy for menopausal women.^[46]^ Long-term use may lead to side effects such as nausea, headaches, breast pain, and an increased risk of blood clots and endometrial cancer. Studies have indicated that estrogenic drugs influence OA mainly through their regulation of cartilage and bone metabolism. Specifically, estrogen interacts with chondrocytes via ER-α and ER-β, promoting the synthesis of cartilage matrix and inhibiting matrix metalloproteinase activity, which reduces cartilage degradation.^[47]^ Decreasing estrogen levels in postmenopausal women may result in cartilage degradation and accelerate OA progression. Additionally, estrogen plays a crucial role in bone metabolism, decreasing bone resorption and increasing bone density.^[48]^ Coumestrol, a natural phytoestrogen primarily found in legumes, structurally resembles Estradiol and binds to ER-α and ER-β, mimicking estrogen to regulate physiological responses.^[49]^ Its estrogen-like activity is believed to help regulate female hormone levels and provide protective effects against postmenopausal symptoms, osteoporosis, cardiovascular health, and hormone-related cancers.^[50,51]^ Despite its potential health benefits, more clinical trials are necessary to confirm its efficacy and safety. In summary, we hypothesize that estrogenic drugs may interact with *HIPK2* to inhibit chondrocyte degradation and slow OA progression. Biotin and Phenobarbital also demonstrate strong interactions with *HIPK2*. Biotin, or vitamin B7, is an essential component of coenzymes involved in various metabolic processes, particularly fatty acid synthesis, amino acid metabolism, and gluconeogenesis.^[52]^ It is often used for preventing or treating thinning hair or brittle nails.^[53]^ Additionally, biotin plays a role in regulating blood glucose and promoting glucose metabolism, making it a candidate for adjuvant therapy in diabetes mellitus.^[54]^ Biotin is generally safe, with few reported adverse effects, even at high doses. Phenobarbital, a long-acting barbiturate, depresses central nervous system activity by enhancing γ-aminobutyric acid receptor activity, acting as a sedative and anticonvulsant.^[55]^ It is included in the World Health Organization’s list of essential medicines and is widely used to treat various types of epilepsy, particularly generalized tonic-clonic seizures and focal epilepsy. In some instances, phenobarbital is also employed as an anesthetic aid for surgery or to sedate patients suffering from insomnia.^[56]^ Recent studies suggest that phenobarbital may be beneficial in treating severe alcohol withdrawal syndromes^[57]^ though the precise mechanism remains unclear.^[58]^ However, it is important to note that phenobarbital can cause significant drowsiness, fatigue, and cognitive decline, and long-term use may lead to drug dependence.

This study has several limitations. First, all conclusions were derived from publicly available transcriptomic datasets and in silico analyses, without in vitro or in vivo experimental validation. Second, the association between *HIPK2*, candidate genes, and ECM-related pathways is correlative and does not establish causality. Third, immune infiltration analysis, CIBERSORT deconvolution, and molecular docking are all indirect computational approaches with inherent methodological assumptions. Therefore, the present findings should be regarded as hypothesis-generating. Future studies should validate these observations through gain- and loss-of-function experiments, chondrocyte functional assays, ECM-related phenotyping, and in vivo OA models.

## 5. Conclusion

Overall, our integrative analyses suggest that *HIPK2* is associated with OA and may be linked to ECM-related dysregulation in FC. *ZNF638*, *DMXL1*, and *VEZT* emerged as candidate genes associated with *HIPK2* expression and ECM-receptor interaction pathway activity. In addition, molecular docking identified several compounds as preliminary candidates for future validation. These findings provide a basis for hypothesis generation and future mechanistic investigation, but direct experimental studies are required to determine causality and therapeutic relevance.

## Author contributions

**Conceptualization:** Chunli Bai, Yuxiang Zhang, Bo Gao, Wenli Fan, Gang Ma.

**Data curation:** Chunli Bai, Yuxiang Zhang, Bo Gao, Wenli Fan, Gang Ma.

**Formal analysis:** Chunli Bai, Yuxiang Zhang, Bo Gao, Wenli Fan, Gang Ma.

**Funding acquisition:** Chunli Bai, Yuxiang Zhang, Gang Ma.

**Writing – original draft:** Chunli Bai, Yuxiang Zhang, Gang Ma.

**Writing – review & editing:** Chunli Bai, Yuxiang Zhang, Gang Ma.
